# Severe Chronic Pain Following Retroperitoneal Hemorrhage in a COVID-19 Patient: Amelioration with a Topical Pain Cream

**DOI:** 10.1089/pmr.2020.0102

**Published:** 2020-10-13

**Authors:** Jennifer Winegarden, Daniel B. Carr, Victoria Pike

**Affiliations:** ^1^ Department of Hospice Medicine, The Medical Team Hospice, Livonia, Michigan, USA.; ^2^ Department of Public Health, Tufts University School of Medicine, Boston, Massachusetts, USA.

*To the editor:*

Morbidity related to severe acute respiratory syndrome coronavirus 2 (SARS-CoV-2) infection continues to rise and includes acute and chronic pain.^1^ Multiple etiologies include viral-induced myalgia, neuropathic pain, tissue hypoxia-ischemia,^2^ and rhabdomyolysis. Iatrogenic causes include anticoagulation resulting in bleeding in patients who receive anticoagulation empirically based on elevated inflammatory markers.^3^

We recently treated a 68-year-old woman with nasopharyngeal-swab positive coronavirus disease 2019 (COVID-19) with acute-to-chronic pain after a left retroperitoneal bleed. She was hospitalized with fever, cough, myalgia, D-dimer of 3600 ng/mL and hemoglobin 12.6 gm/dL. She remained stable and was discharged with enoxaparin 90 mg injections twice daily while continuing on aspirin 81 mg daily.

Four days later the patient developed back and leg pain with weakness and syncope. The patient's primary care physician was notified who suspected a common nerve injury. When symptoms worsened overnight the patient went to the emergency room where a low-back X-ray and lower extremity Doppler were negative. An MRI obtained 10 days after starting enoxaparin revealed a 900-cc left-sided retroperitoneal hematoma that was surgically drained but with no improvement in her lower extremity pain and weakness. After two months of in-patient rehabilitation the pain continued with 3–4/10 pain intensity at rest and 10/10 with movement. An electromyography (EMG) study four months after surgery revealed severe acute denervation of the femoral nerve, left lumbosacral plexopathy, distal left lower extremity entrapment neuropathy, and right femoral neuropathy.

The patient's pain was treated with Kadian 15 mg by mouth twice daily, to which was added morphine 2 mg IV every two hours as needed (PRN), alternating with oxycodone 5 mg q six hours PRN; gabapentin 100 mg three time daily (TID) orally; cyclobenzaprine 5 mg q eight hours PRN with a pain of 7/10 with activity. Increases of gabapentin to 300 mg twice daily (BID) and Biofreeze topical spray only reduced her pain to 3–5/10 at rest and 6/10 with activity. Because adequate pain control with acceptable side effects was difficult to achieve, tissue concentrations in the area of pain were targeted with a topical compounded cream of ketamine 15%, clonidine 2%, and gabapentin 4%/1 mL Lipoderm cream; applying 1.0 mL TID in the left femoral nerve cutaneous distribution. Ketamine was chosen for *N*-methyl-d-aspartate receptor (NMDAR) antagonism, clonidine for hyperpolarization of the afferent neuron membrane, and gabapentin continued topically for neuronal N-type calcium channel inhibition. Topical lidocaine was not included; our experience relegates topical lidocaine to use in mild-to-moderate pain.^4^ This augmented regimen reduced the pain to 0–2/10 during the day, despite activity, although after a day of extended activity the patient found oxycodone 5 mg PRN helpful for sleep.

Treatment of pain during intercurrent COVID-19 including neuropathic pain from a retroperitoneal hematoma, as in this patient, may be facilitated by multimodal therapy including low doses of topical agents.

## Abbreviations Used

COVID-19: coronavirus disease 2019
NMDAR: *N*-methyl-d-aspartate receptor
PRN: when necessary
SARS-CoV-2: severe acute respiratory syndrome coronavirus 2
TID: three times daily
